# Detection of lyssavirus antigen and antibody levels among apparently healthy and suspected rabid dogs in South-Eastern Nigeria

**DOI:** 10.1186/s13104-018-4024-z

**Published:** 2018-12-22

**Authors:** Ukamaka U. Eze, Ernest. C. Ngoepe, Boniface M. Anene, Romanus C. Ezeokonkwo, Chika Nwosuh, Claude T. Sabeta

**Affiliations:** 1 Department of Veterinary Medicine, Faculty of Veterinary Medicine, University of Nigeria, Nsukka, Enugu State Nigeria; 2 OIE Rabies Reference Laboratory, Agricultural Research Council-Onderstepoort Veterinary Institute, Onderstepoort, South Africa; 3 Department of Veterinary Parasitology and Entomology, Faculty of Veterinary Medicine, University of Nigeria, Nsukka, Enugu State Nigeria; 4 National Veterinary Research Institute, Vom, Plateau State Nigeria; 5 Department of Veterinary Tropical Diseases, University of Pretoria, Onderstepoort, 0110 South Africa

**Keywords:** Lyssavirus, Dogs, Antigen, Antibodies, DFA, ELISA

## Abstract

**Objectives:**

Domestic dogs are the main reservoir of rabies virus (RABV) infection in Nigeria, thus surveillance of rabies in dog populations is crucial in order to understand the patterns of spread of infection and ultimately devise an appropriate rabies control strategy. This study determined the presence of lyssavirus antigen in brain tissues and anti-rabies antibodies in sera of apparently healthy and suspected-rabid dogs slaughtered for human consumption at local markets in South-Eastern Nigeria.

**Results:**

Our findings demonstrated that 8.3% (n = 23) of brain tissues were lyssavirus positive and 2.5% (n = 25) of sera had rabies antibody levels as percentage blocking of 70% and above correlating with a cut-off value ≥ 0.5 IU/mL in the fluorescent antibody neutralization test. There was an inverse correlation between lyssavirus positivity and rabies antibody levels confirming that infected individuals most often do not develop virus neutralizing antibodies to the disease. The low percentage of rabies antibodies in this dog population suggests a susceptible population at high risk to RABV infection. These findings highlight a huge challenge to national rabies programs and subsequent elimination of the disease from Nigeria, considering that majority of dogs are confined to rural communal areas, where parenteral dog vaccination is not routinely undertaken.

**Electronic supplementary material:**

The online version of this article (10.1186/s13104-018-4024-z) contains supplementary material, which is available to authorized users.

## Introduction

The aetiologic agent of rabies is rabies virus (RABV); a non-segmented single-stranded RNA virus and member of the genus *Lyssavirus* (family *Rhabdoviridae*, order *Mononegavirales*). In Africa and Asia, approximately 99% of human rabies cases are dog-mediated [1], making rabies a disease of significant public and veterinary health threat, albeit that it is preventable through parenteral vaccination. Consumption of dog meat is common in some specific regions of Nigeria. This practice is a potential risk for the importation of RABV-infected dogs into the dog markets for slaughter from different regions and countries. Consequently, handling and processing of such meat exposes meat-handlers to potentially RABV-infected dogs [2–4]. Previous studies demonstrated that some of the slaughtered dogs were infected with lyssaviruses [2–4]. Despite these observations, neither surveillance nor a tangible rabies control strategy has been formulated and deployed by local governments.

For routine diagnosis of RABV, detection of viral antigen in the central nervous system tissues is generally undertaken by the use of the direct fluorescent antibody test (DFA) [5], applying either a fluorescein isothiocyanate (FITC) labelled anti-rabies monoclonal (Mab) or Polyclonal antibody (Pab) to an acetone-fixed composite brain smear [6]. Virus neutralizing antibodies (NAs) are a key component of the adaptive immune response against viral pathogens including RABV [7], and neutralization assays are currently the gold standard test for assessing NAs. The Fluorescent antibody virus neutralization test (FAVNT) [8] and the rapid fluorescent foci inhibition test (RFFIT) [9] are both recommended by the World Health Organization (WHO) and the World Organization for Animal Health (OIE) [6, 10]. In addition, ELISA assays can be considered as alternatives to neutralization assays, especially for use in epidemiological surveys where large numbers of samples are involved and in laboratories where cell culture facilities with appropriate containment for neutralization assays may be lacking [6, 11]. In Nigeria, previous studies in dog populations have involved serological analysis [12–15]. However, there is dearth of information on the antigenic status of dogs whose sera were examined. This study was therefore undertaken to assess the presence of lyssavirus antigen in parallel to levels of anti-glycoprotein antibodies among apparently healthy and rabies-suspect dogs slaughtered for meat consumption in local markets in South-Eastern Nigeria.

## Main text

### Methodology

#### Study area

A total of 278 brain tissues and accompanying serum samples were collected from both apparently healthy and rabies-suspect dogs at (dog) markets, restaurants and veterinary clinics in three South-Eastern States of Nigeria (Enugu, Ebonyi and Anambra) (latitudes 7°07′N and 3°90′N and longitudes 6°51′E and 8°30′E) (see map of study area, Additional file 1) between October 2015 and July 2016.

#### Specimen collection

The criteria used in this study, to identify rabies-suspect dogs were based on history and typical clinical presentation [16–18]. Dogs without history of rabies and not displaying clinical signs of the disease prior to slaughter were considered apparently healthy. For each dog, brain sample was collected and stored in a leak-proof container. Also 3 mL of blood was collected from the cephalic vein into a vacutainer, allowed to clot, and then centrifuged at 3000 rpm for 15 min (Centromix, P. Selecta; Barcelona), to separate the serum. All samples were stored at − 20 °C until tested.

#### Rabies virus antigen detection

Brain tissues were subjected to the DFA test [5] with a lyophilized, adsorbent anti-rabies nucleocapsid monoclonal globulin labeled with FITC (Fujirebio^®^, USA) at the National Veterinary Research Institute (NVRI, Jos, Nigeria), according to the manufacturer’s guidelines. Brain samples were retested using anti-rabies nucleocapsid monoclonal globulin labeled with FITC (BIORAD^®^) at the Rabies Unit, Onderstepoort, South Africa, (an OIE Rabies Reference Laboratory). The presence of lyssavirus antigen was confirmed by observation of typical clusters of apple green fluorescing aggregates of Negri bodies under UV fluorescence.

#### Rabies virus neutralizing antibody detection

Assessment of antibody levels was conducted at the Rabies Unit, Onderstepoort, South Africa on heat-inactivated sera (at 56 °C for 30 min) [8]. The BioPro ELISA kit (BioPro, Prague, Czech Republic) based on a blocking ELISA protocol was used to detect the presence or absence of anti-glycoprotein rabies antibodies. To confirm the results from The ELISA test, the FAVNT was done on selected samples and their end-point titres established. The BioPro ELISA kit has been validated by several global OIE Rabies Reference Laboratories to support as a potential alternative to seroneutralization assays for monitoring oral vaccination [11]. The percentage blocking (PB%) of each serum sample was calculated in the serum samples, according to the manufacturer’s recommendations and interpreted as per OIE and WHO recommendations [19, 20]. Serum samples with PB < 40% are considered negative, while serum samples with PB ≥ 40% but < 70% are considered positive for RABV antibodies when antibody levels are ≤ 0.5 IU/mL on VN test. Serum samples with PB ≥ 70% are considered positive when antibody levels are ≥ 0.5 IU/mL on the FAVNT [19, 20]. In addition, the FAVNT was performed on all serum samples with PB ≥ 40% to establish their end-point titres [8]. The three microplates used in the ELISA assay showed that the optical density (OD) of the positive and negative controls of all the microplates met the validation criteria indicated by the manufacturer.

#### Statistical analyses

Serological results were summarized using descriptive statistics on the GraphPad prism statistical package version 5.2 for Windows (GraphPad Software, La Jolla California USA, http://www.graphpad.com). Chi square test was used to test association between the presence of lyssavirus antigen and health status and presence of lyssavirus antigen versus location. Odds ratio was used to determine the strength of association between the variables. Correlation and regression analysis were done to test relationship between ELISA and FAVNT.

### Results

**Fig. 1:**
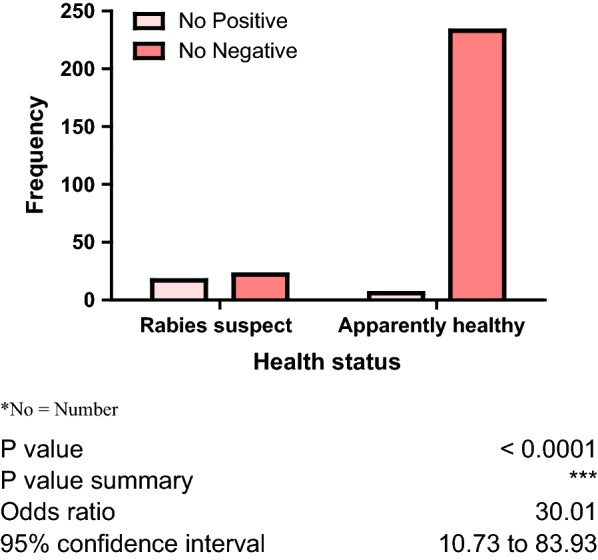
Association between the presence of lyssavirus and health status of 278 dogs sampled in South Eastern Nigeria

The prevalence of lyssaviruses in the study population was 8.3% (n = 23). Three (4.8%) of the 62 samples collected from Anambra State were lyssavirus positive, compared to two (3.1%) out of 65 samples collected from Ebonyi State, whereas 18 (11.9%) out of 151 samples collected from Enugu State tested positive for rabies virus antigen. There was no association (P > 0.05) between distribution of lyssavirus antigen and state of origin of the dogs sampled. However, there was a strong association (P > 0.05) between the presence of lyssavirus antigen and health status of dogs as dogs suspected to be rabid showed significantly higher number of lyssavirus positive brain tissues than the apparently healthy dogs, Fig. 1.

Of the 260 serum samples collected, only 252 serum samples were included in the final analysis as eight of the samples were lipaemic and thus not suitable for testing. The PB distribution of the ELISA results showed that 233 (92%), 14 (5.6%) and 5 (2.0%) lie in the ranges between − 1 to 39%, 40 to 69% and ≥ 70%, respectively Table 1.

**Table 1:** Summary of the ELISA results from the three States in South Eastern Nigeria where dog sera samples were collected for *Lyssavirus* antibodies assessment

States	Percentage blocking	Anambra	Ebonyi	Enugu	Total
		N (Pos %)	N (Pos %)	N (Pos %)	N (Pos %)
Locality of origin of samples					
Ranges of Percentage blocking	− 1 to 39	56 (93.3)	59 (95.2)	118 (90.8)	233 (92.5)
	40 to 69	4 (6.7)	1 (1.6)	9 (6.9)	14 (5.6)
	70 and above	0 (0)	2 (3.2)	3 (1.5)	5 (2.0)
Total number collected from each state		60	62	130	252

*N* (*pos %*) = number positive

**Fig. 2:**
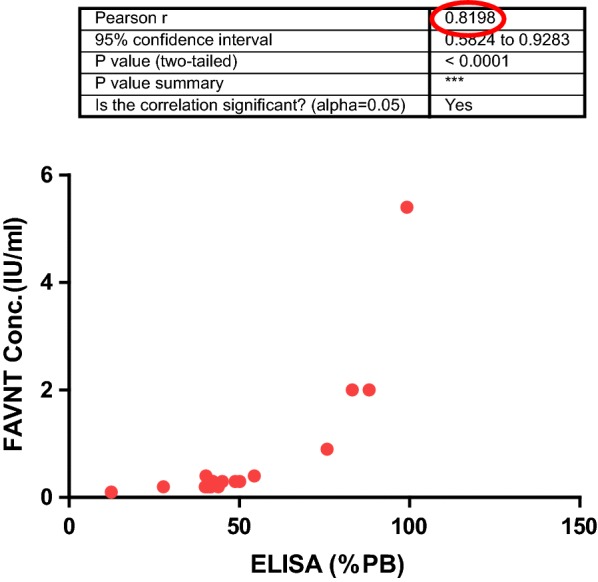
Correlation between ELISA and FAVNT

There was no correlation between the PB of the serum and the presence of lyssavirus antigen as the brain tissues that had PB of ≥ 70% were all DFA negative. However, there was a strong correlation between the blocking ELISA and the FAVNT results, Fig. 2.

All the samples that have PB of ≥ 70% were above 0.5 IU/mL (with FAVNT), a cut-off value indicating adequate seroconversion of a dog following vaccination or a possible exposure to a lyssavirus within phylogroup1 [21, 22].

### Discussion

This study was designed to determine the presence of lyssavirus antigen and quantify rabies anti-glycoprotein antibody in a cross-section of dogs at point of slaughter in local markets in South-Eastern Nigeria. The findings established presence of lyssavirus antigen in both apparently healthy and rabies-suspect dogs in South-Eastern Nigeria in approximately 8.3% of the dogs tested (*n *= 23). However, 2.5% (*n *= 6) were from dogs that were apparently healthy at the time of slaughter. These findings corroborated earlier reports that detected lyssavirus antigen in the brains of apparently healthy dogs slaughtered at local markets in Nigeria sometimes originating from different regions of the country. Earlier reports indicated prevalence of between 1.58 and 5% [2, 3, 23, 24]. Two independent groups isolated RABV from apparently healthy dogs following several passages in cell culture [25] and in mice and demonstrated these isolates are different from vaccinal strains [26].

The high numbers of suspect-rabid dogs [17 (43.6%)] probably reflects that the true burden of rabies in Nigeria is grossly underestimated, but also strengthens the argument for the need to enhance the knowledge of the public health hazards of rabies amongst dog marketers in South-Eastern Nigeria. Generally speaking, positive dog rabies cases confirmed from the (dog) markets are not included in the annual rabies data reported to the OIE. As a result, rabies is highly underreported, with likelihood that rabies is at least hundred fold higher than officially reported. Furthermore, national governments do not seem to prioritize rabies but findings from this study may inform policy change, to ensure that rabies is placed high on the priority list.

Dog markets present areas with a potential risk of rabies outbreaks and transmission to humans. This makes dog markets particularly significant and strategic because the people selling dogs in Nigeria bring dogs from different parts of the country, including illegal importations [27, 28].

The ELISA results demonstrated that majority of the dogs slaughtered for consumption lack detectable levels of rabies antibodies in their sera, an indication of the high susceptibility of this dog population to RABV infection. Only 2% of the dogs’ population possessed antibodies against rabies indicating very low herd immunity. This finding further suggest that little or no dog vaccination takes place in the rural areas and is consistent with observations from other parts of the country [12, 13]. RABV-specific antibodies detected in some apparently healthy wild terrestrial carnivores and bats probably suggest exposure of these animals to lyssaviruses including RABV [29–32].

The results of FAVNT on the small panel of serum samples corroborated the results of the blocking ELISA, which is consistent with findings from Wasniewski et al. [11] who concluded that the ELISA is reliable and reproducible, and could be useful in rabies antibody detection especially during sero-surveys. This is particularly important for a Nigerian setting where containment laboratory for FAVNT is lacking. Rabies-infected dogs had no detectable rabies antibodies confirming observations that infected individuals most often do not develop VNAs [33]. Failure of the host (dog) to produce a protective response that successfully resolves the infection or reduces the severity of the disease could be explained by the immune evasion triggered by pathogenic lyssaviruses [34]. Only two of the dogs infected with RABVs had PB% of 54% [0.4 IU/mL] and 48% [0.3 IU/mL] respectively, which were well above the first cut off value (of 40%), which suggests that these dogs may be in the advanced stage of the disease at the time of slaughter. Appropriate studies on the pathogenicity, cell mediated immunity and humoral responses in a natural canine host (dog) may help to shed more light on the mechanisms of immune responses to infection in this species.

### Conclusion

In conclusion, this study has demonstrated that there is some likelihood of some dogs destined for the markets to be rabies-infected and a large number of rabies naive dogs exist in rural South-Eastern Nigeria. In addition, the importation of unvaccinated dogs, slaughter and processing of dog meat in these markets contributes to the cycle of neglect resulting in rabies being under-reported in Nigeria. Consequently, this may make elimination of dog-mediated human rabies deaths difficult to achieve by the set target of 2030. Therefore, mass dog vaccination campaigns in rural regions of Nigeria should be enhanced in order to successfully eliminate dog rabies.

## Limitation

Although in this study, we used the DFA which is the gold standard test for rabies diagnosis recommended by WHO, however other confirmatory tests such as mouse inoculation test and reverse-transcriptase polymerase chain reaction were not used to confirm negative results.

## Additional file

**Additional file 1.** Geographical location of South Eastern Nigeria and the States involved in the study. Map of the study area.

## Abbreviations

ELISA: enzyme-linked immunosorbent assay
FAVNT: fluorescent antibody virus neutralization test
RABV: rabies virus
DFA: direct fluorescent antibody test
RNA: ribonucleic acid
FITC: fluorescein-isothiocyanate
Mab: monoclonal antibody
Pab: polyclonal antibody
NAs: neutralizing antibodies
RFFIT: rapid fluorescent foci inhibition test
WHO: World Health Organization
OIE: World Organization for Animal Health
NVRI: National Veterinary Research Institute
UV: ultra violet
PB: percentage blocking
OD: optical density
VN: virus neutralization
