# Supplementary figures and images for: Detection of lyssavirus antigen and antibody levels among apparently healthy and suspected rabid dogs in South-Eastern Nigeria
Source: BMC Res Notes. 2018 Dec 22;11:920. doi: 10.1186/s13104-018-4024-z (PMC6303872; doi:10.1186/s13104-018-4024-z)

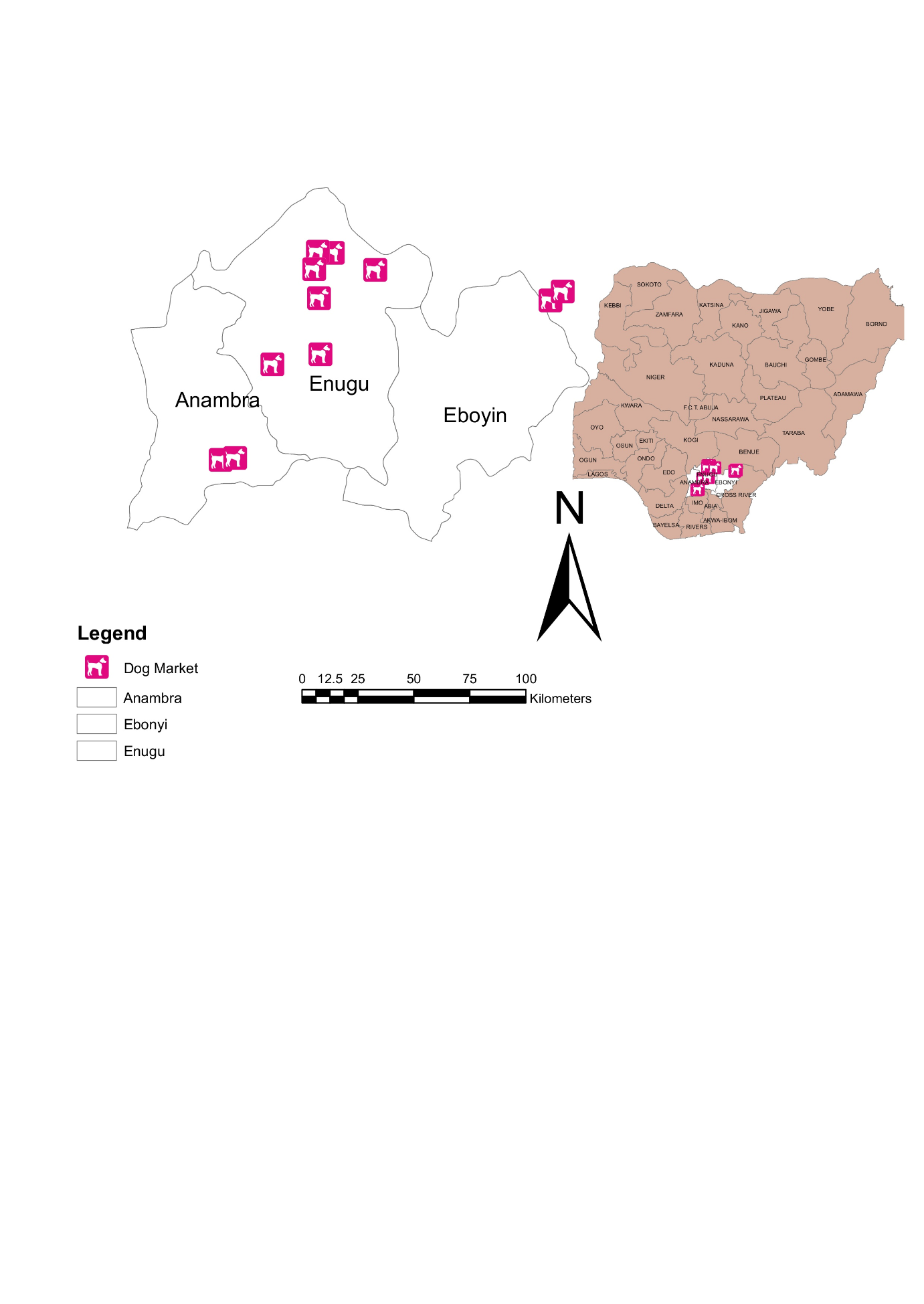


**Additional file 1**

Supplement: Supplementary file 1 — Additional file 1. Geographical location of South Eastern Nigeria and the States involved in the study. Map of the study area. [file 13104_2018_4024_MOESM1_ESM.docx]
